# Editorial: Nuclear morphology in development and disease

**DOI:** 10.3389/fcell.2023.1267645

**Published:** 2023-08-08

**Authors:** Tanmay P. Lele, Daniel L. Levy, Krishnaveni Mishra

**Affiliations:** ^1^ Department of Biomedical Engineering, College of Engineering, Texas A&M University, College Station, TX, United States; ^2^ Artie McFerrin Department of Chemical Engineering, College of Engineering, Texas A&M University, College Station, TX, United States; ^3^ Department of Translational Medical Sciences, Texas A&M University, Houston, TX, United States; ^4^ Department of Molecular Biology, University of Wyoming, Laramie, WY, United States; ^5^ Department of Biochemistry, School of Life Sciences, University of Hyderabad, Hyderabad, Andhra Pradesh, India

**Keywords:** nucleus, lamins, lamina, mechanics, size, shape, morphology

The size and shape of the nucleus vary in different cell types and can change during developmental progression, cellular differentiation, and aging. Aberrant nuclear morphology is associated with a multitude of disease states, including cancers and laminopathies. While some mechanisms responsible for determining nuclear morphology have been elucidated (Lele et al., 2018; Kalukula et al., 2022), much less is known about how nuclear morphology becomes altered in disease and whether nuclear morphology directly impacts cell function. The goal of this Research Topic was to begin to address the functional significance of nuclear morphology. Seven interesting papers were published on a diverse array of Research Topic related to the nucleus, ranging from therapeutic normalization of enlarged nuclei in cancer to the scaling relationship between nuclear size, cell size, and genomic content in frog erythrocytes, to the mathematical prediction of nuclear shapes in cultured mammalian cells.

Niide et al. report a careful characterization of nuclear size, cell size and genomic content, with an emphasis on intra-as well as inter-species scaling relationships. They found distinct scaling patterns within species, compared to the patterns between species. Specifically, the study revealed variations in the contributions of cell area and genomic content to nuclear size determination, with genomic content having a more significant impact in amphibians, and cell size having a more significant impact in non-amphibians. The scaling relationships may ultimately help shed light on how cell size, nuclear size, and genome size might have co-evolved during evolution.

The correlation between nuclear size and cell size may be due, at least in part, to transport through nuclear pores (Deviri and Safran, 2022). Facilitated transport of cargo into the nucleus through nuclear pores occurs through binding of cargo to specialized cytoplasmic transport receptors called importins. While transport is their primary function, depending on the cellular context, importins also have other functions. In their review, Damizia et al. discuss these alternate functions which range from regulation of the mitotic spindle to preventing phase separation of toxic proteins in neurons.

The control of nuclear size is also important from the point of view of human pathologies such as cancer. Nuclear size is frequently seen to increase in diverse cancers, which has led to its use as a diagnostic tool (Singh and Lele, 2022). Schirmer et al. propose an intriguing hypothesis based on a recently published drug screen that sought to rectify nuclear size changes—that is, increase nuclear size if the size was reduced in a particular cancer cell type, and decrease it if it increased in a particular cancer cell type. Drugs that rectified nuclear size changes also tended to reduce migration and/or invasion in a range of assays. Based on these data, the authors propose a novel four-part hypothesis. In its essence, the hypothesis is that nuclear size changes contribute to metastatic spread and invasion in cancer, and that drugs that rectify these changes could serve as potent therapies. Schirmer et al. raise the exciting possibility of translating nuclear size-targeted therapies into the clinic.

In addition to changes in nuclear size, nuclear shape can also become substantially altered in cancer and in the natural process of aging. Das et al. performed a detailed characterization of both nuclear shape and size among neurons in the mouse brain. The study revealed variations in nuclear size and shape across different regions in the brain, related to age, and in neurons in a mouse model of Alzheimer’s disease. The shape and size heterogeneities were found to be region specific. Whether these changes are contributory to disease (this possibility was discussed in the context of cancer above) or whether they are purely biomarkers of aging and disease is a Research Topic for further exploration.

In addition to nuclear size changes, nuclear shape abnormalities also abound in human diseases and in natural processes such as aging, but the underlying mechanisms remain unclear. Dickinson and Lele formulated a mathematical model to calculate nuclear shapes in cultured cells in various contexts, which were then compared to experimentally measured shapes. Close agreement between predicted and experimental shapes supports a previously proposed geometric principle of nuclear shaping (Dickinson et al., 2022; Dickinson and Lele, 2023): the excess surface area of the nuclear lamina that manifests in the form of folds/wrinkles permits a wide range of highly deformed nuclear shapes under the constraints of constant surface area and constant volume. When the lamina becomes tensed (unwrinkled), a limiting nuclear shape is reached, which can be predicted entirely from these geometric constraints alone for a given cell shape. Whether excess surface area of the nuclear lamina for a given nuclear volume (size) becomes altered significantly in diseases requires further exploration.

One consequence of the limiting shapes of nuclei that are attained in flattened cells or in cells squeezing through confining environments, is that the actomyosin cortex abutting the nuclear surface can pressurize the nucleus to such an extent as to cause rupture (Denais et al., 2016; Hatch and Hetzer, 2016). Envelope rupture can cause nuclear contents to leak out into the cytoplasm, exposing chromatin to cytoplasmic proteins and promoting DNA damage (Nader et al., 2021; Shah et al., 2021). Unsurprisingly, mechanisms exist in cells to repair the nuclear envelope after rupture (Halfmann et al., 2019). Borah et al. review how specialized proteins present in the inner nuclear membrane, the so-called LEM domain proteins (Lap2-emerin-Man1 proteins), recruit ESCRT (endosomal sorting complex required for transport proteins) to repair the ruptured nuclear envelope. Rounding off this Research Topic of papers, Chmielewska et al. trace chromosome composition in spermatogenic cells. Their studies reveal new insight into the process of hybridogenesis, a reproductive strategy in hybrid tadpoles, demonstrating that hybridogenesis can result in reduced fertility due to incomplete elimination of chromosomes during prespermatogenesis.

In sum, this Research Topic of articles addresses diverse aspects of nuclear morphology—ranging from mechanisms of size and shape determination to nuclear morphology-targeted therapies for human diseases. Combining accurate nuclear size and shape measurements in different cell types and disparate diseases, together with measurement of functional consequences such as nuclear rupture, and computational modeling of nuclear mechanics, can substantially enhance the diagnosis and treatment of human diseases.
